# The beneficial effect of combination therapy with sulfasalazine and valsartan in the treatment of ulcerative colitis

**DOI:** 10.17179/excli2021-3370

**Published:** 2021-02-05

**Authors:** Fereshteh Asgharzadeh, Atieh Yaghoubi, Seyedeh Elnaz Nazari, Alireza Hashemzadeh, Seyed Mahdi Hasanian, Amir Avan, Ali Javandoost, Gordon A Ferns, Saman Soleimanpour, Majid Khazaei

**Affiliations:** 1 Department of Physiology, Faculty of Medicine, Mashhad University of Medical Sciences, Mashhad, Iran; 2 Student Research Committee, Faculty of Medicine, Mashhad University of Medical Sciences, Mashhad, Iran; 3 Antimicrobial Resistance Research Center, Bu-Ali Research Institute, Mashhad University of Medical Sciences, Mashhad, Iran; 4 Department of Microbiology and Virology, Faculty of Medicine, Mashhad University of Medical Sciences, Mashhad, Iran; 5 Department of Medical Biochemistry, Mashhad University of Medical Sciences, Mashhad, Iran; 6 Neurogenic Inflammation Research Center, Mashhad University of Medical Sciences, Mashhad, Iran; 7 Metabolic Syndrome Research Center, Mashhad University of Medical Sciences, Mashhad, Iran; 8 Department of Medical Genetics and Molecular Medicine, Faculty of Medicine, Mashhad University of Medical Sciences, Mashhad, Iran; 9 Brighton & Sussex Medical School, Division of Medical Education, Falmer, Brighton, Sussex BN1 9PH, UK

**Keywords:** combination therapy, valsartan, angiotensin II receptor blocker, colitis, inflammation

## Abstract

Inflammatory bowel disease (IBD) is defined by the chronic inflammation of the digestive tract. Ulcerative colitis is one of the most prevalent chronic IBDs. The increase in the mucosal expression of angiotensin II (AT-II) in colitis suggests a possible role of AT-II in colitis-associated inflammation. Here, we examined the potential therapeutic effects of combination therapy regarding valsartan (Val), as an AT-II receptor blocker, with sulfasalazine (SSZ) in a murine colitis model. DSS induced colitis was initiated by the administration of dextran sodium sulfate (DSS) in male C57BL/6 mice for 1 week. Val (160 mg/kg/day, gavage) was given on the third day and continued for seven days. SSZ (100 mg/kg/day) was used as reference drug and also used in combination in one group (Val; 160 mg/kg/day and/or SSZ; 100 mg/kg/day). Colonic mucosal inflammation was evaluated clinically, biochemically, and histologically. The disease activity index in DSS-treated mice, including weight loss, stool consistency, and rectal bleeding, were significantly lower in the group of mice receiving the combination of valsartan and sulfasalazine compared to the DSS-treated group. Valsartan and sulfasalazine treatment was associated with a lower reduction in colon length, diminished colon weight, and high sensitivity C-reactive protein level in mice with DSS-induced colitis. Valsartan and sulfasalazine also reduced markers of oxidative stress after DSS administration. Our findings demonstrate the anti-inflammatory and anti-fibrotic activities of a combination therapy with sulfasalazine and valsartan in experimentally induced colitis, indicating its value as a potential therapeutic option for the treatment of colitis.

## Introduction

The inflammatory bowel disease (IBD) is a common inflammatory digestive condition that is categorized into two major types: ulcerative colitis (UC) (Okawada et al., 2011[27]) and Crohn's disease (CD) (Beniwal-Patel and Shaker, 2019[6]). IBD is a multifunctional disorder of uncertain etiology. However, one or more of the following factors are involved, immune system dysfunction (caused by genetics or environmental factors), changes in normal gastrointestinal flora, mucosal barrier injury, and oxidative stress, which play a key role in this condition (Beniwal-Patel and Shaker, 2019[6]). UC is a common type of IBD with high prevalence globally (Beniwal-Patel and Shaker, 2019[6]; Kaplan, 2015[19]). Chronic mucosal inflammation, colicky abdominal pain, bloody diarrhea, fatigue, nausea, and weight loss are characteristic of ulcerative colitis (Beniwal-Patel and Shaker, 2019[6]; Flynn and Eisenstein, 2019[11]). Patients with UC have a higher risk of developing colorectal cancer (CRC) (Tajti et al., 2019[33]). Chronic colon inflammation is the major underlying cause in the progress of colitis-associated cancer, which is stimulated by oncogenic signaling pathways, resulting in unregulated cell proliferation in the intestinal tract (Tajti et al., 2019[33]). There is still a need for safe and effective treatment for patients with IBD (Flynn and Eisenstein, 2019[11]). The majority of existing colitis therapies target inflammatory and immune reactions in patients (Flynn and Eisenstein, 2019[11]). Sulfasalazine is the first-line pharmacotherapy for ulcerative colitis that has partially absorbed into the jejunum, which then passes through coliforms to the colon, where it is changed to sulfapyridine and its active form named 5-ASA (Flynn and Eisenstein, 2019[11]; Plosker and Croom, 2005[29]). Other IBD therapies include (i) corticosteroids, immunomodulatory drugs such as Purinethol (Mercaptopurine), Azasan (azathioprine), and Trexall (methotrexate), and (ii) tumor necrosis factor-alpha (TNFα) inhibitors such as Remicade (infliximab) and Humira (adalimumab) (Flynn and Eisenstein, 2019[11]). However, the adverse effects of long-term therapy in patients with IBD, limit the effectiveness of treatment. Sulfasalazine has several potential side effects, including pancreatitis, hepatitis, rash, and agranulocytosis (Moum, 2008[24]). In addition, corticosteroids raise the risk of osteoporosis and suppression of the immune system. Furthermore, Azasan and Purinethol show different side effects such as pancreatitis, hepatotoxicity, and blood disorders, while Trexall can induce myelosuppression, pulmonary fibrosis, and hepatotoxicity (Amidon et al., 2015[2]; Kumar and Mutlu, 2015[22]). 

The renin-angiotensin system (RAS) is a crucial agent in regulating the vascular tone and the balance of salt and fluids (Garg et al., 2012[12]). This system is a multi-component cascade with renin as a rate-limiting enzyme that delivers angiotensinogen to AT-I, which is broken down to AT-II by angiotensin-converting enzyme (Asgharzadeh et al., 2018[5]). Additionally, to hormonal activity, AT-II has pro-inflammatory properties which may play an inflammatory role in IBD patients (Garg et al., 2020[13]). The latter is the principal RAS effector that is binding to angiotensin receptors (AT1 and AT2) present in several forms of cells, including bowel epithelial cells and mucosal immune cells (Garg et al., 2020[13]). The angiotensin II type 1 receptor (AT1R) is also found in the stroma of intestinal fibroblasts (Raka et al., 2018[30]). It has been shown that AT-II, acting via an AT1R, induces reactive oxygen species and activates the kappa B (NF-κB) nuclear factor (Ruiz-Ortega and Ortiz, 2005[31]). Derangements of the RAS are associated with different cardiovascular and kidney diseases, while their role in digestive disorders such as inflammatory bowel disease is unclear (Garg et al., 2012[12]). Circumstantial evidence suggests the participation of the RAS in the pathogenesis of colitis (Shi et al., 2016[32]). According to one report, the colonic mucosal level of the AT-I and AT-II increases in CD patients with active inflammation (Khajah et al., 2016[20]). Furthermore, knock-out mice with AT1R or angiotensinogen deletions, show less severe colitis in comparison to the wild-type mice with colitis (Shi et al., 2016[32]). Thus, the AT1 receptor blocker may result in the attenuation of inflammatory symptoms associated with colitis. Here we aimed to examine the beneficial effects of valsartan in combination with sulfasalazine as well as an AT-II receptor blocker, in the treatment of the experimental subjects with colitis.

## Materials and Methods

### Materials

Sulfasalazine powders were obtained from Iran Hormone Co. (Tehran, Iran). Valsartan, hematoxylin, eosin (H&E), and other reagents as the catalase (CAT), superoxide dismutase (SOD), malonyl dialdehyde (MDA), and total thiol reactions were obtained from Sigma Co. (Saint Louis, MO).

### Induction of colitis and study design

C57BL/6 male mice, 7 to 8 weeks old (purchased from Pasteur Institute, Tehran, Iran) were housed in a 12-hour light/dark cycle room, acclimatized 7 days before the experiments, and divided into five groups randomly (n = 6 in each group), including the control group, colitis group, sulfasalazine (SSZ) as positive control groups (100 mg/ kg/day), Val (160 mg/kg/day) group and Val + SSZ (160 mg/kg/day and 100 mg/kg/day, respectively) (Khajah et al., 2016[20]; Valero et al., 2020[34]). All mice experiments were carry out in accordance with the guideline for Care and Use of Laboratory Animals from Mashhad University of Medical Sciences.

**Figure 1:**
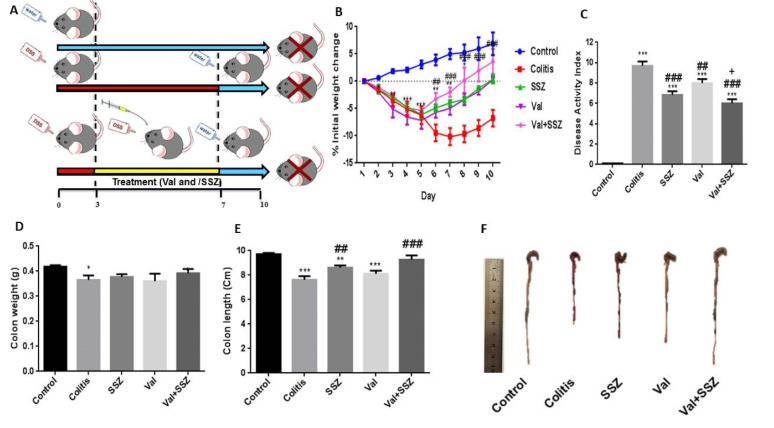
Schematic representation of experimental procedure (A), and the clinical symptoms of murine colitis model such as changes in body weight (B); Highest changes in disease activity index (DAI) during the experimental period (C); colon weight (D); colon length (E), and macroscopic image of colon (F). Data are presented as the mean ± SEM. ***P<0.001, compared to control group. (### P<0.001 and ## P<0.01 compared to colitis group. + P<0.05 compared to SSZ group).

Colitis in the mice was induced by the oral intake of 1 % DSS (w/v, dissolved in drinking water) for 1 week in all the experimental groups (except the control group) from the first to the seventh day. The control group was given distilled water during the experimental period. The other groups were administered the various interventions orally for 7 consecutive days at the aforementioned doses once daily (from the third to the seventh day) (Figure 1A(Fig. 1)).

### Evaluation of the degree of colitis

During the treatment period, bodyweight changes, visible stool consistency, and rectal bleeding were measured daily. The disease activity index (DAI) (Hamer et al., 2010[16]) was scored based on the parameters described in Table 1(Tab. 1). The mice were euthanized after treatment, and the whole colon was collected (from the caecum to the rectum). The length of the entire colon was measured, and the weight reported after a longitudinal opening and flushing with PBS solution. The colon section in the mice was divided into three parts (the proximal, middle and distal parts). For the histological analyses, the distal colon parts were fixed in 10 % formaldehyde. The proximal and middle sections of the colon were separated into three parts for subsequent molecular and biochemical analyses.

### Histological analysis 

The distal colon was fixed at room temperature in 10 % formalin overnight and then coated in paraffin. The colon tissues were sectioned at 5 mm thickness and then treated with H&E, as well as Masson's trichrome stains. Images were attained using an Olympus microscope with 40 and 100 magnifications. The proportion of fibrotic tissue regions stained with Masson trichrome was measured as fibrosis. The fibrosis region was evaluated using ImageJ tools. Then, the colon tissue damage was scored according to the histopathological index (Table 2(Tab. 2)).

### Determination of oxidative stress index

The colon samples were weighed and homogenized on ice with PBS. Then, the homogenate was centrifuged at 4 °C, for 10 min at 3000 rpm. The supernatant was collected and stored at −70 °C for the evaluation of oxidant/antioxidant indicators including MDA, total thiol, SOD, and CAT activity (Bordoni et al., 2019[7]).

#### Determination of MDA 

The MDA content was calculated as a measure of lipid peroxidation in colon sample. Colon tissue was homogenized, and a homogeneous aliquot was applied to the thiobarbituric acid reactive material. The absorption of the mixture was measured against a blank using a spectrophotometer at 535 nm, and the MDA quality of the samples was analyzed using a standard curve. 

#### Determination of total thiol groups (SH) 

The dithio nitro benzoic acid (DTNB) assay was used to determine the total thiol content of the colonic homogenates. The criterion for plotting the standard curve was taken as reduced glutathione. Supernatants were incubated in 0.1 L Tris-EDTA buffer with DTNB (pH 8.6). In the next step, this mixture was incubated at 25 °C for 10 min., and the absorption was spectrophotometrically measured at 412 nm. The quality of GSH was measured using a regular curve.

#### Determination of SOD

Evaluation of SOD activity was performed based on the development of superoxide dismutase by pyrogallol auto- and the inhibition of MTT conversion to formazan. DMSO was then used to dissolve formazan and to create stable colors. The absorbance was eventually read to be 570 nm (Hashemzehi et al., 2020[17]).

#### Determination of CAT

The catalase activity was calculated at 240 nm by the spectrophotometer according to the rate of decomposition of hydrogen peroxide (Aebi, 1974[1]).

### Statistical analysis 

The data was stated as mean ± Standard Error of Mean. The one-way ANOVA test followed by LSD as a post-hoc test was used to compare various groups. The rate p<0.05 was considered as statistically significant rate. 

## Results

### Valsartan improves the clinical symptoms of murine model of chronic colitis 

Mice are highly susceptible to DSS treatment and exhibit significant rectal bleeding and weight loss within 3-4 days of receiving DSS. The 1 % DSS intake resulted in a loss of body weight. Figure 1B(Fig. 1) shows that 1 % of DSS treatment results in rapid weight loss. This result confirms that the loss of body weight and subsequent recovery are important reproducible markers for the occurrence and extent of colitis caused by DSS. The body weight of animals in the untreated group increased steadily, while the body weight of the colitis group decreased significantly and sustainably compared to the control group. In addition, the body weights of animal in the groups receiving SSZ, Val, or Val +SSZ significantly improved from day 5 to day 10 (Figure 1B(Fig. 1)). To determine the severity of colitis, an initial increase in body weight, rectal bleeding, and stool consistency were observed to produce a score for the disease activity index. The changing DAI score for different groups can be seen in Figure 1C(Fig. 1) after seven days of treatment. For the mice treated with DSS, it was significantly higher (maximum DAI score of 9.7±0.42, Figure 1C(Fig. 1)) compared to the control group (p<0.001). The DAI score in the group of Val (maximum DAI score 8±0.40, Figure 1C(Fig. 1)) and Val + SSZ (maximum DAI score 6±0.40, Figure 1C(Fig. 1)) was significantly lower in the group of colitis, respectively p<0.01 and p<0.001. Also, the DAI score was significantly lower in the Val + SSZ group (maximum DAI score of 6±0.40) compared to the SSZ group (maximum DAI score of 6.85±0.34; p<0.05) (Figure 1C(Fig. 1)).

### Valsartan ameliorates the colon damage induced by the DSS-treated mice

Loss of body weight and rectal bleeding are also related to thinning and colon shortening. Colon measurement in the DSS-treated group indicated a decreased colon weight relative to the control group (p<0.05). All treatedgroups show increased colon weight compared to the group receiving DSS alone, but this was not significant (Figure 1D(Fig. 1)). The DSS-treated mice colons were 7.63 cm long on average and significantly shorter than the control mice colons, which had an average length of 9.72 cm (p<0.002) (Figure 1E(Fig. 1)). In contrast, the Val treatment attenuated colonic shortening (Val + SSZ →9.27cm; p<0.001 and Val →8.12cm; p>0.05). Moreover, SSZ show similar effects to reduce colonic shortening (length of 8.61 cm) in the colitis caused by DSS. The macroscopic image of the colon is shown in Figure 1F(Fig. 1).

### Valsartan reduced inflammation in the colitis mice models

**Figure 2:**
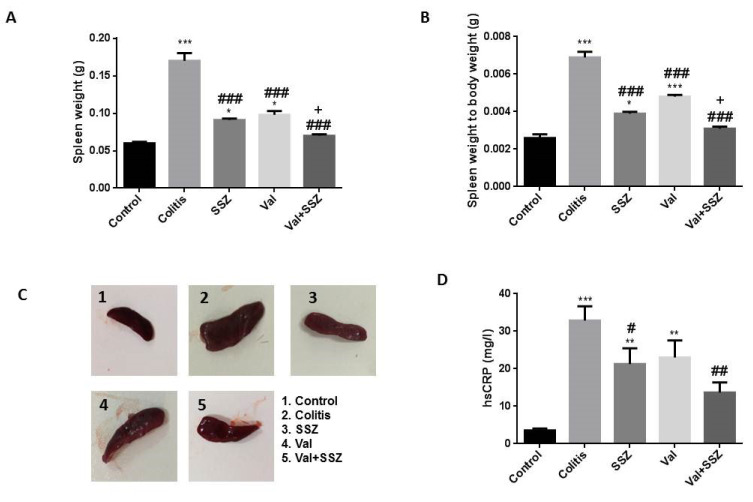
Anti-inflammatory effect of valsartan. (A) Spleen weight; (B) Spleen weight to body weight; (C) Macroscopic image of spleen; and (D) High Sensitivity C-Reactive Protein (hsCRP). Data are presented as the mean ± SEM. (***P<0.001, **P<0.01 and *P<0.05 compared to control group. ### P<0.001 and # P<0.05 compared to colitis group. + P<0.05 compared to SSZ group).

The measurement of spleen weight (Figure 2A(Fig. 2)) and spleen weight to body weight ratio (Figure 2B(Fig. 2)) showed that valsartan in combination with SSZ has been able to reduce these clinical manifestations in the colitis mice models caused by DSS. Compared to colitis animal, the control and treated groups with SSZ and Val significantly show the attenuation of spleen weight and spleen weight to body weight ratio (All p<0.001). Additionally, the spleen weight and spleen weight to body weight ratio in the Val + SSZ group were lower than the SSZ group (p<0.05). These results show that treatment with valsartan in combination with SSZ decreased serum hsCRP levels in the colitis group (p<0.01) (Figure 2D(Fig. 2)). These findings indicate that valsartan protective effects against colitis probably were regulated through the activation of anti-inflammatory reactions.

### Attenuation of the colonic histopathological changes in DSS-treated mice by valsartan

**Figure 3:**
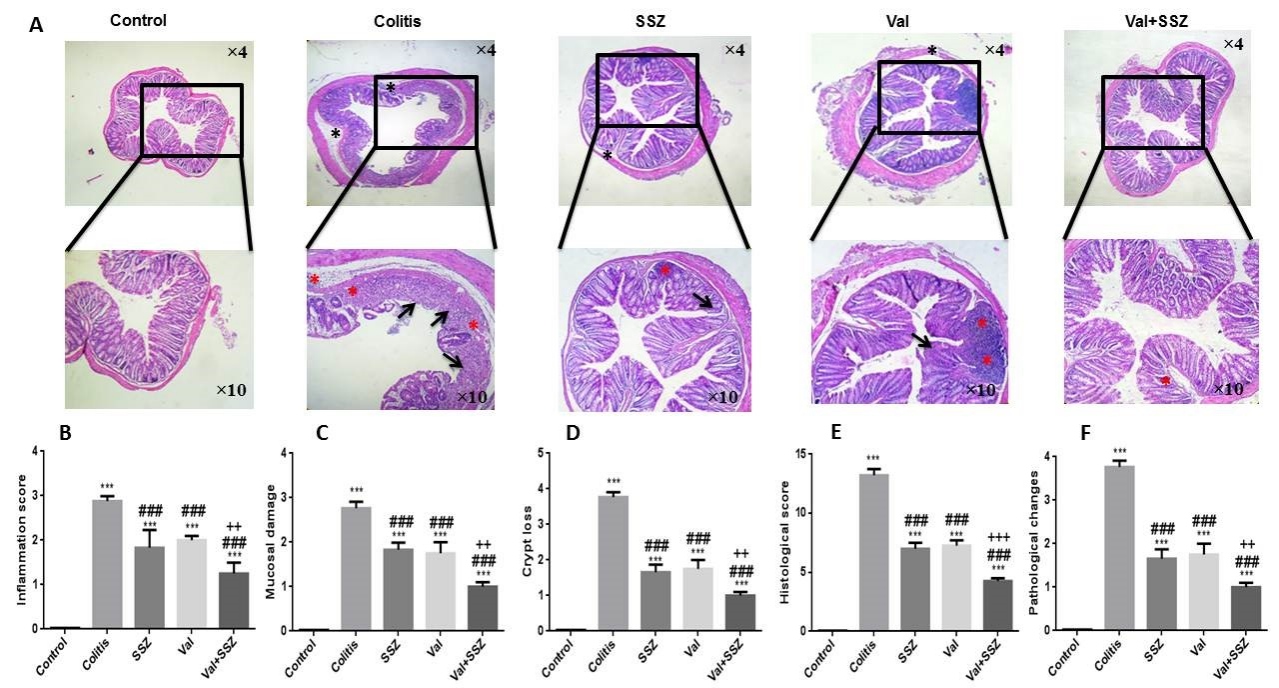
Histopathology of colon tissue. (A) Representative histopathology images of colon with Hematoxylin and Eosin staining (H&E), (B) inflammation score, (C) mucosal damage, (D) crypt loss, (E) histological score, (F) pathological changes. The DSS image is highlighted by evaluated and scored features: red asterisks indicate inflammation, black arrows indicate crypt loss, black asterisks indicate mucosal damage. Data are presented as the mean ± SEM. (***P<0.001 compared to control group, ### P<0.001 compared to colitis group, +++ P<0.001 and ++ P<0.01compared to SSZ group).

The historical index of the colons was investigated via H&E staining (Figure 3A(Fig. 3)). Animal preserved the integrated normal colonic structures in the control group, characteristic of the transparent outer membrane, muscle layer, submucosa, and the mucosa. On the other hand, DSS treatment eliciting deterioration in histological scores (Figure 3F(Fig. 3)), such as mucosa inflammation (Figure 3B(Fig. 3)), mucosal damage (Figure 3C(Fig. 3)), crypt lack (Figure 3D(Fig. 3)), and pathological range change (Figure 3F(Fig. 3)) has significantly higher histological scores as compared to the control group (all parameters p<0.001). However, compared to colitis mice, mice treated with Val, SSZ or in combination showed remarkable improvement in intestinal injury (Figure 3A(Fig. 3)), relief of mucosal inflammation (Figure 3B(Fig. 3)), loss of crypt (Figure 3D(Fig. 3)) and pathological area change (Figure 3E(Fig. 3)), and a significant reduction in histological criteria (Figure 3F(Fig. 3)) (all historical index for all groups: p<0.001). Combination therapy of Val with SSZ improved mucosa inflammation (Figure 3B(Fig. 3)), mucosal damage (Figure 3C(Fig. 3)), crypt lack (Figure 3D(Fig. 3)), pathological range change (Figure 3E(Fig. 3)) (all parameters with p<0.01), and histological criteria (Figure 3F(Fig. 3)) in comparison to the group treated only by SSZ (p<0.001).

### Valsartan inhibits colon fibrosis in chronic colitis of mouse model 

**Figure 4:**
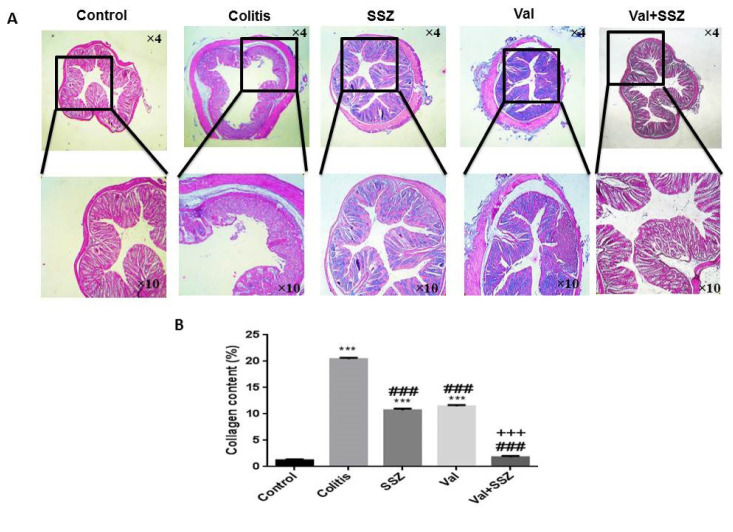
Anti-fibrotic of valsartan. (A) Representative histopathology images of the colon with trichrome staining, and (B) Quantification of collagen in the colon by Image J, Data are presented as the mean ± SEM. (***P<0.001 and **P<0.0 compared to control group. ### P<0.001 compared to colitis group. +++ P<0.001 compared to SSZ group).

Excessive deposition of collagen in the bowels causes fibrosis, which is a crucial pathological stage of colitis. Our findings indicated that DSS increases the fibrosis visualized by Masson's trichrome collagen staining in the colon sample of colitis animal significantly (Figure 4A(Fig. 4)). Valsartan, either alone or in combination with SSZ decreased DSS-induced collagen deposition in the colon (both p<0.001) (Figure 4B(Fig. 4)). The collagen content in the Val + SSZ group displayed a significant decrease in comparison with the SSZ alone group (p<0.001). 

### Valsartan attenuated oxidative stress in colitis

**Figure 5:**
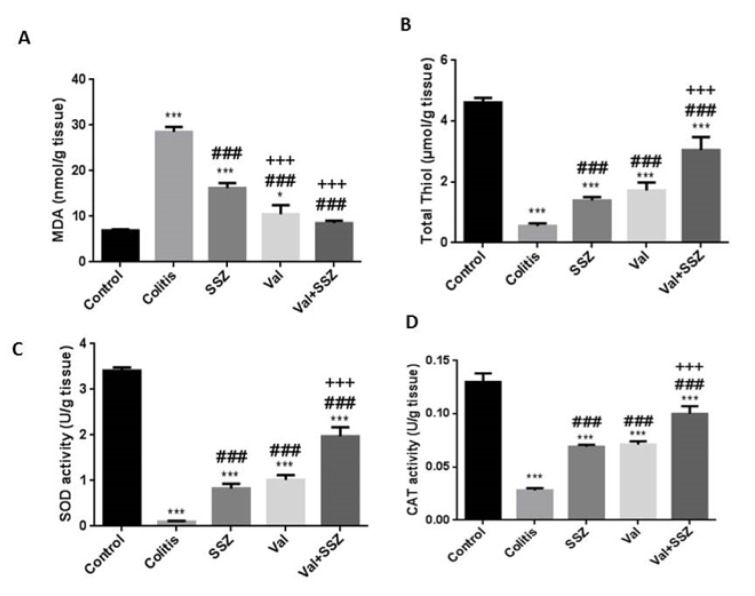
Colonic oxidant and antioxidant activities in mice following the induction of colitis by DSS administration. (A) Malondialdehyde (MDA), (B) Total thiol, (C) Superoxide dismutase (SOD), (D) Catalase (CAT). Data are presented as the mean ± SEM. (***P<0.001 and *P<0.05 compared to control group. ### P<0.001 compared to colitis group. +++ P<0.001 compared to SSZ group).

In comparison to the DSS-treated mice, our findings showed that the administration of Val and Val + SSZ significantly decreased the levels of MDA as an end product of lipid per- oxidation (Figure 5A(Fig. 5)), while total thiol (Figure 5B(Fig. 5)), superoxide dismutase (Figure 5C(Fig. 5)) and catalase (Figure 5D(Fig. 5)) were increased as the antioxidant markers (p<0.001 for all the markers). Compared to SSZ alone, the use of valsartan (Val) with SSZ reduced the oxidant index and significantly increased the antioxidant indicators (p<0.001).

## Discussion

From an evolutionary point of view, the RAS is a system of pro-inflammatory impact on different tissues. It has paracrine and autocrine actions in addition to the endocrine effects. Currently, angiotensin antagonists are generally used in the treatment of hypertension. Valsartan is known as angiotensin II receptor blocker (ARBs) that can bind to the AT1R and displace AT-II from this receptor. This medication has been utilized in humans to treat mild to moderate blood pressure (Kirk, 1999[21]). In the present study, we used the AT1R blocker instead of an Angiotensin-converting Enzyme (ACE) inhibitor to avoid the mechanism that is based on AT-II formation by the cell chymase. We used the DSS mouse model, that is known as the most common colonic inflammation model. This method is widely used to evaluate the human inflammatory bowel disease model (Eichele and Kharbanda, 2017[9]). DSS is toxic to epithelial cells and seems to be phagocytozed by macrophages, interfering with the connection between intestinal lymphocytes and epithelial cells (Ni et al., 1996[26]). In the intestinal tract, DSS causes mild to moderate inflammation, marked by bloody diarrhea. Our result demonstrates that valsartan alone or in combination with sulfasalazine is able to attenuate the inflammatory symptoms including loss of body weight, shortening of the colon length, and colonic tissue damage in the DSS-induced mouse colitis model. The DAI scores in the valsartan-treated group were lower than the DSS group. Several articles have suggested that different members of the ARB family have anti-colitis effects (Arab et al., 2014[3]; Guerra et al., 2015[15]; Liu et al., 2016[23]; Nagib et al., 2013[25]; Okawada et al., 2011[27]). Similar to the previous studies, the results of the current study also verify the protective effect of ARBs in pro-inflammatory diseases such as colitis and colorectal carcinogenesis (Arumugam et al., 2015[4]; Okawada et al., 2011[27]; Shi et al., 2016[32]), Alzheimer's disease (Ongali et al., 2014[28]), breast cancer (Chen et al., 2013[8]), and lung adenocarcinoma (Wilop et al., 2009[35]). Furthermore, olmesartan administration to mice before inducing the ulcerative colitis model had a similar or even better effect as compared to sulfasalazine (Nagib et al., 2013[25]). The hsCRP levels are commonly used in the follow-up of IBD patients. CRP is synthesized mainly in the hepatocytes, while extra-hepatic output has also been proved. The upregulation of protein is largely due to an increase in interleukin-6 (IL-6) and, to a lesser degree, an increase in IL-1β and tumor necrosis factor-α (TNF-α) (Henriksen et al., 2008[18]). Shi et al. showed that AT-II infusion exacerbates colitis, while endogenous RAS blockade ameliorates it (Shi et al., 2016[32]). Overall, these data strongly indicate that the RAS is a colitogenic factor, and activation of RAS could promote colitis. Pro-inflammatory cytokines were reduced in the human biopsies from patients with IBD who were on the ARB therapy relative to those who had not undergone the ARB therapy. These findings indicate that RAS activation encourages colitis independently of hypertension. AT-II seems to promote colonic mucosal inflammation by promoting the apoptosis of the intestinal epithelial cells and responses to TH17 in the development of colitis (Shi et al., 2016[32]). Previous studies indicate that losartan and candesartan used in a DSS colitis model may protect against colonic apoptosis, epithelial degradation, and pro-inflammatory cytokine abundance significantly. These findings suggest that the pathogenesis of DSS colitis can be implicated in the relationship between AT-II and AT1R. The use of specially formulated AT1R elements with low oral absorption such as deschloro-losartan known as a novel therapeutic approach for the treatment of IBD that may have great potential (Okawada et al., 2011[27]). 

It has been shown that the oxidant/antioxidant balance is disrupted during colon inflammation caused by epithelial cell membrane damage (Yao et al., 2010[36]). The production of reactive oxygen species (ROS) in the colon of patients suffering from IBD is significantly increased (Hamer et al., 2010[16]). Our result demonstrates that valsartan can significantly reduce inflammation and balance oxidant/antioxidant by suppressing hs-CRP and increasing CAT activity. In addition, valsartan is able to inhibit the ROS generation and modulates the CAT activity in the oxidative stress induced by inflammation in the human CRC cells (El-Azab et al., 2016[10]). Moreover, MDA is significantly decreased in colitis, while the thiol level is increased considerably after the olmesartan therapy (Nagib et al., 2013[25]). Furthermore, telmisartan treatment of rats (5 mg/kg) subject to treatment with acetic acid, had significantly reduced MDA levels and increased the IL-10 production. Additionally, after treatment with telmisartan, macroscopic damage, the number of ulcers and histopathological processes were reduced (Guerra et al., 2015[15]). A study showed that losartan treatment improved colitis in the wild-type mice, suggesting a colitogenic function for the endogenous RAS (Okawada et al., 2011[27]). However, there is a need for further research on the scientific and pathological basis of valsartan protection effects in clinical administration for patients with colitis. Another study showed that losartan was able to reduce the effects of 2,4,6-trinitrobenzene sulfonic acid (TNBS) colitis in mice. Thus, it probably prevented the intestinal epithelial cell apoptosis by increasing the Bcl-2/Bax ratio and reducing proapoptotic caspase-3 activity (Liu et al., 2016[23]). Pretreatment of rats with telmisartan reduced TNBS-induced colitis decreased the disease severity and suppressed the inflammatory response by attenuating the function of TNF-α, prostaglandin E2, and Myeloperoxidase and restoring IL-10. Telmisartan similarly reduced oxidative stress, as demonstrated by lipid peroxide suppression and nitric oxide, as well as improved glutathione, total anti-oxidant ability, and SOD and GPx activities (Arab et al., 2014[3]). Regarding the DSS-induced colitis in mice, AT-II receptor blockers significantly enhanced clinical, histological ratings, and epithelial cell apoptosis. In addition, ARB therapy had substantially reduced mRNA of TNF-α, IL-1β, and IL-6 (Okawada et al., 2011[27]). Efficient anti-fibrosis therapies appear to be a major unmet need in the patients with IBD (Gordon et al., 2018[14]). Therefore, we assessed the effect of this therapeutic inhibitor on the fibrosis induced by the colitis. Our findings suggest that co-treatment with valsartan suppresses the expression of pro-fibrotic agents, which is consistent with other research works, since it clearly indicates a correlation between the inflammation and fibrosis. Garg et al. assessed the RAS effect on the intestinal inflammation and fibrosis according to these findings (Garg et al., 2020[13]). RAS mediates fibrosis in the cultures of human cells that is expressed in the intestine and disrupted by intestinal inflammation, and agents that control this function such as ARB are associated with improved outcomes of diseases (Garg et al., 2020[13]).

In conclusion, our findings show that some of the parameters associated with intestinal inflammation can be decreased by the combination therapy using sulfasalazine and valsartan. This experiment indicates a potential anti-inflammatory effect in colitis for valsartan through modulation of the immune system. However, these results require more investigation to evaluate the therapeutic potential for using ARB in the IBD.

## Notes

Saman Soleimanpour and Majid Khazaei (Department of Physiology, Faculty of Medicine, Mashhad University of Medical Sciences, Mashhad, Iran; E-mail: Khazaeim@mums.ac.ir) contributed equally as corresponding authors.

## Conflict of interest

The authors declare no conflict of interest.

## Figures and Tables

**Table 1:**
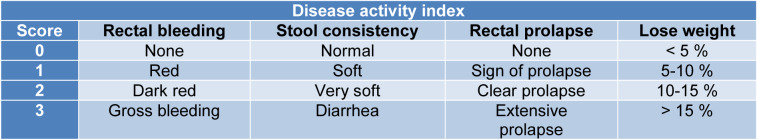
The disease activity index scoring parameters

**Table 2:**
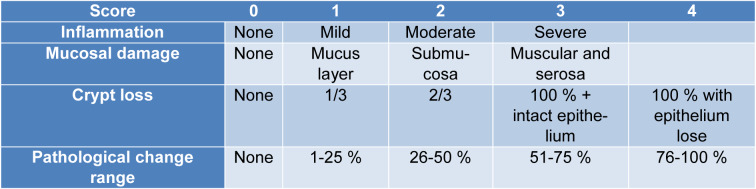
Scoring parameters of colonic histological changes
